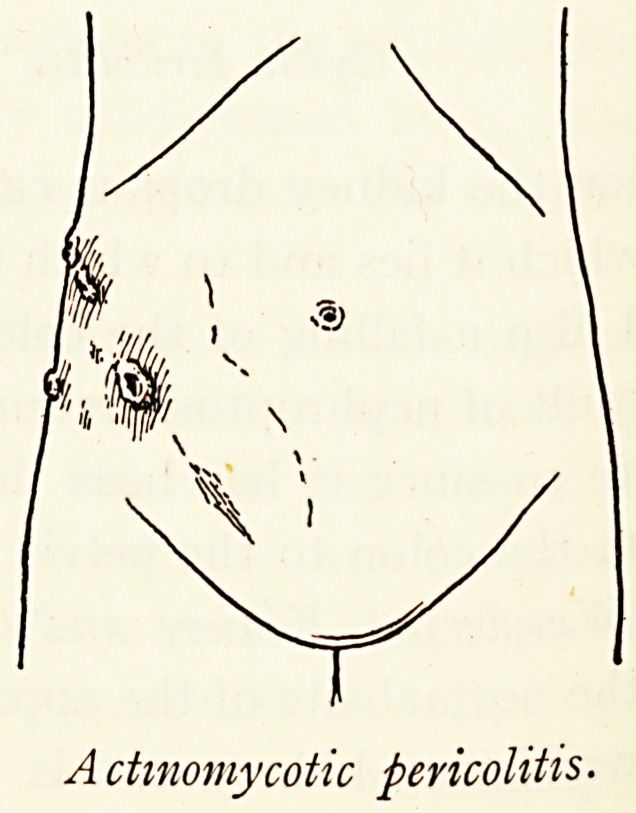# Pericolitis

**Published:** 1913-12

**Authors:** T. Carwardine

**Affiliations:** Honorary Surgeon to the Bristol Royal Infirmary


### PERICOLITIS.

BY

T. Carwardine, M.S., F.R.C.S.,

*Honorary Surgeon to the Bristol Royal Infirmary.*

---

Observations on the living indicate that in persistent and local fæcal stasis there is inflammation of the covering of the colon, associated with exudate. With marked fæcal impaction a localised peritonitis may result, and peritoneal adhesions may persist after the passage of the obstruction. In cases where there is ulceration a corresponding inflammation may ensue in the mesentery or cellular interval. This is exemplified in the meso-appendix, which is often thickened and contracted opposite the site of constriction of the appendix (meso-appendicitis), and in meso-sigmoiditis following ulceration of the sigmoid flexure.

*Mesoappendicitis.*

Whilst it would appear that inflammation takes the chief share in the formation of pericolic adhesions, it is probable that some of the bands are acquired in reaction to stress and strain, and that some are of congenital origin. It must be borne in mind that the original inflammatory process may subside and the remote consequences persist, and this occurs not uncommonly from appendicitis. If in such a case the appendix be removed without dealing with the remote mechanical results the operation may not give complete relief. Hence it has become desirable to make the incisions and examinations sufficiently extensive to deal with the remote as well as the local trouble. Thus, in operations for supposed chronic appendicitis, it is essential to examine the lower six inches of the ileum, and the ascending colon as far as the hepatic flexure.

Perhaps it will be as well to consider the subject under several headings :—

*Pericolitis from Appendicitis.*—This may be represented by a few simple adhesions of little importance, or by bands which mechanically interfere with peristalsis or even cause obstruction. Although the inflammatory peritoneal attack may clear up, the persistence of wide-spread adhesions indicates its original extent. The commonest condition is when the appendix is bound down, and sometimes angulated as well as fixed; and

*Appendicular adhesions.*

*Pericolitis from appendicitis.*

when the fixation is retro-cæcal the mechanical impediment to the cæcum is great, tethering it down, and giving rise to painful peristalsis and sometimes ballooning. Moreover, if it be true that waves of peristalsis begin at the tip of the appendix and a short distance from the end of the ileum—and synchronously reach the cæcum—it is obvious that adhesions interfere with the normal cycle. The position of the pericolic bands depends

*Ileal loop from adhesions to cæcum.*

*Pericolitis persisting after appendicectomy.*

in part on that of the appendix, whether free, pelvic, retro-cæcal or paracolic. When the appendix projects downwards and inwards the pelvic organs in the female may become involved, or inflammatory bands may bind the lower ileum down to the pelvis, which is the only variety of kink of the ileum which I have met with at present. Rarely the ileum, some distance from its termination, may become adherent to the cæcum, forming a distinct and fixed loop. When the appendix is paracolic the bands are mostly to the outer side of the colon, where the omentum may also be attached; and the convolutions of the colon may be bound together. In one case recently operated upon I found distinct cysts which had developed in the iliac fossa amongst the adhesions.

*Jackson's Membrane.*—The importance of this has only recently been recognised, and there are all grades of transition, from mere adhesions to the well-developed condition. Typically speaking, Jackson's membrane consists of a thin film covering the ascending colon as by a thin veil, slightly vascularised, freely movable over the surface of the subjacent bowel, and capable of being cut with the scissors, thus exposing the colon beneath. It is continuous with the right border of the omentum

*Pericolic films.*

*Jackson's membrane.*

and sometimes with the anterior layer of a transverse meso-colon, in which case the colon is often re-duplicated upon itself, "double-barrelled." The importance of this membrane becomes the greater because its central fibres may be thickened, so compressing the ascending colon and causing dilatation of the cæcum, or its upper fibres may form a strong band obstructing the bowel at the hepatic flexure.

*Lane's Kink.*—Of the importance of this there can be no doubt in certain cases, although its origin is explained in various ways. In one case I found that its release completely cured a patient of very chronic intestinal obstruction for which she had been admitted several times. The kink is near the lower part of the ileum, produced by a flat band attached to the lower border of the bowel above and the wall of the pelvis below. The

*Pericolitis and Ileal kink.*

cases which I have seen have suggested an inflammatory rather than a mechanical origin, and the kink of the ileum has been in a downward direction. I have never seen the ileum drawn upwards, even allowing for the displacement of the cæcum at the time of the operation. There is, moreover, some evidence in favour of its congenital origin. Anyway, it is frequently met with associated with pericolitis.

*Megaduodenum.*—The explanation of this condition was for some time obscure, but is possibly forthcoming in the presence of some sort of obstruction to the bowel lower down ; not only those impediments previously indicated, but more particularly from a duodeno-jejunal kink—a band constricting the bowel at that junction or at the position of the superior mesenteric artery. The influence of the stasis induced by kinks in the lower bowel in the production of *duodenal and gastric ulcers* has yet to be fully determined by more extended search for them when those ulcers are present.

*Appendix Dyspepsia.*—It is possible that this term has become necessary from giving too great a prominence to the dyspeptic symptoms of appendicitis, and assigning them to the wrong organ. In the absence of adhesions, or a very unusual position of the appendix, the term may be a very uncertain one. My impression is that when the appendix is tethered there may be cæcal distress ; when the tip of the omentum is adherent, then dyspeptic symptoms are the more marked. When the appendix has a very abnormal position the symptoms may be

*Paracolic appendix causing gastric symptoms.*

*Paracolic appendix causing biliary symptoms.*

modified : thus I have known the symptoms of gastric ulcer closely simulated by a paracolic appendix which passed up into the transverse meso-colon, with adhesions to the gall-bladder ; and a mimicry of attacks of biliary colic caused by a retro-peritoneal appendix, the tip of which was close to the common duct.

*Meso-Sigmoiditis and Sigmoid Pericolitis.*—As the sigmoid flexure is a natural reservoir, the stasis in which is influenced by habits and by anatomical variation, its walls are subject to pressure, infection, and sometimes diverticula ; and the meso-sigmoid to chronic inflammation. As a result of diverticula peri-sigmoid abscesses may form, and cancer may have its origin therein. When the meso-sigmoid is involved it undergoes scarring and contracture, becomes adherent to the parietal peritoneum (Toldt's line), and frequently causes the left Fallopian tube or ovary to be drawn up into the scar tissue. This condition will probably receive more attention in the future, as it is the cause of definite symptoms at times, and it may be associated with the similar inflammatory trouble in the right iliac fossa.

*Wandering Kidney and Pericolitis.*—In a recent article I have shown that extreme mobility of the kidney may be a cause of pericolitis, with cystic spaces between the pericolic bands.

*Cystic pericolitis from wandering kidney.*

When the kidney drops it causes a descent of the fascial plane in which it lies and to which it is always adherent in such cases, and also a falling of the colon and peritoneum. Pericolitis as a result of nephroptosis requires more attention given to it, the more so since it has been demonstrated that lymphatics pass from the colon to the pelvis of the kidney.

*Wandering Kidney and Chronic Appendicitis.*—Tenderness at the normal site of the appendix is frequently elicited in cases of wandering kidney and is usually diagnosed as appendicitis.

The removal of the appendix in these cases will not always cure the patient, nor will the fixation of the kidney alone do so. Consequently it has been my practice for some time to remove the appendix and fix the kidney at one operation, with an improvement in the results. Moreover, I am disposed to note

*Wandering kidney*
*h appendicular symptoms.*

*Pericolitis*
*from cholecystitis.*

and treat any pericolitis present at the time. Why a loose kidney should cause symptoms of chronic appendicitis is not quite clear, although venous congestion from pressure and traction may play a part. In obscure cases the only diagnostic aid in differentiation which I have been able to obtain is the following: When appendicular tenderness is caused by a loose kidney, that tenderness disappears or greatly diminishes when the kidney is temporarily replaced.

*Tuberculous pericolitis.*

*Actinomycotic pericolitis.*

*Pericolitis from other Conditions.*—Pericolitis at the hepatic flexure is an almost constant association of gall-stones with cholecystitis, although the intestinal stasis may only be present during the acute attack, the subsequent adhesions impede the colon less here than elsewhere. It is also a natural outcome of ileo-cæcal tuberculosis, actinomycosis, and inflammation of Meckel's diverticulum.

The recognition of the baneful influences of pericolitis has been one of the latest additions to the knowledge of the art of healing.

## Figures and Tables

**Figure f1:**
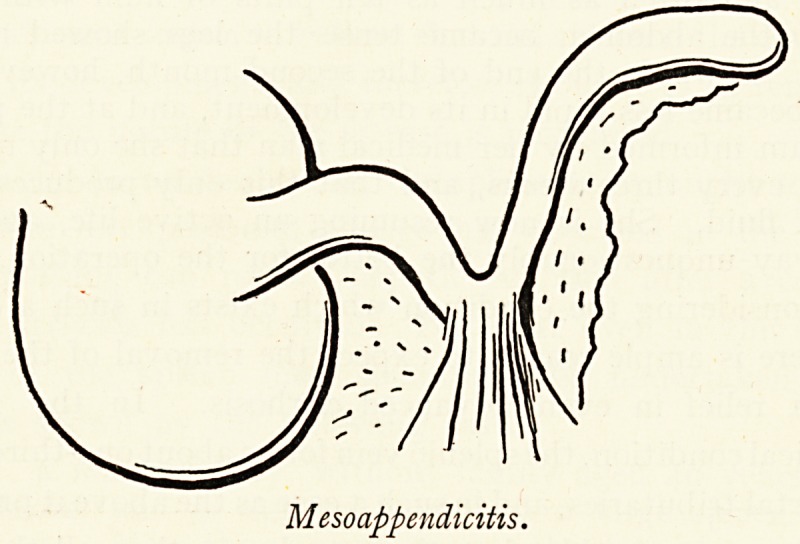


**Figure f2:**
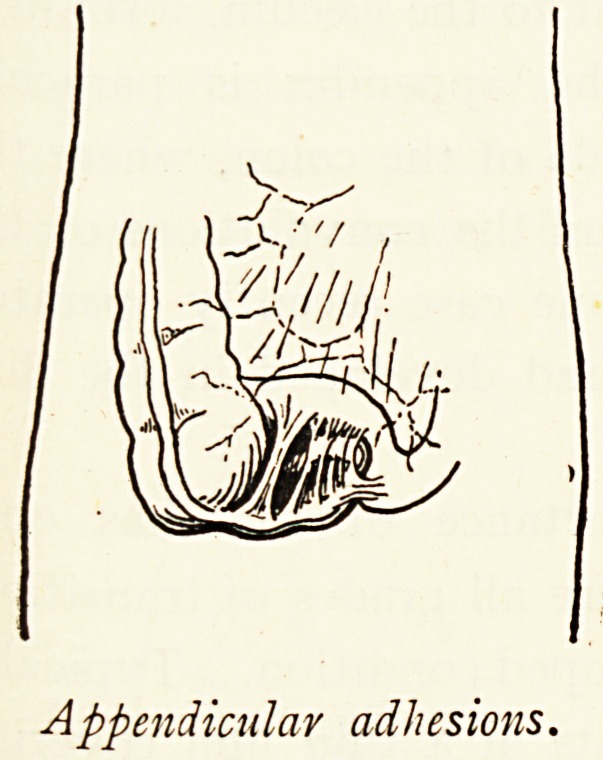


**Figure f3:**
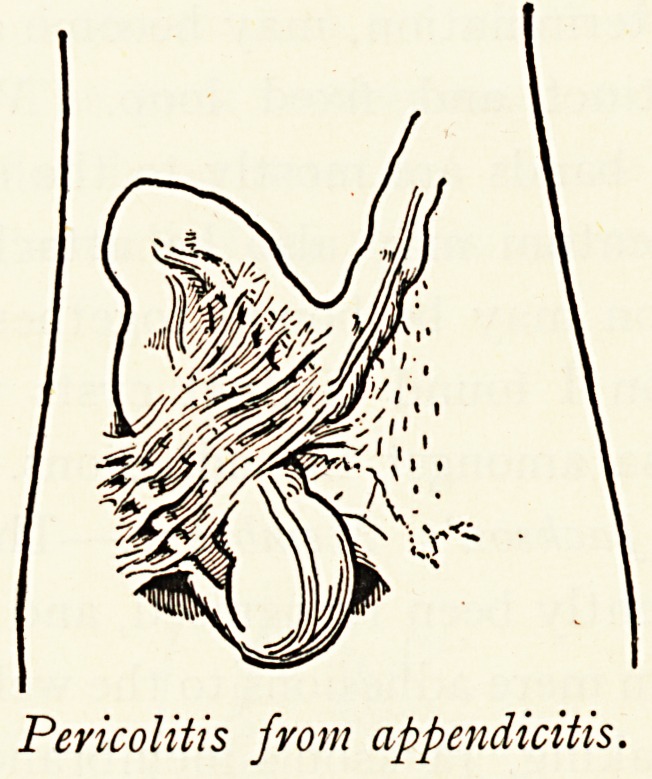


**Figure f4:**
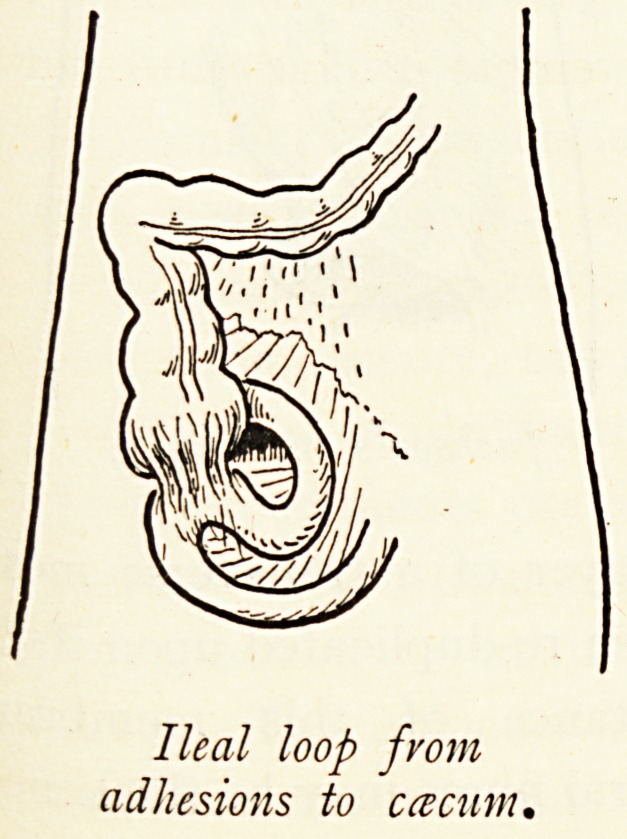


**Figure f5:**
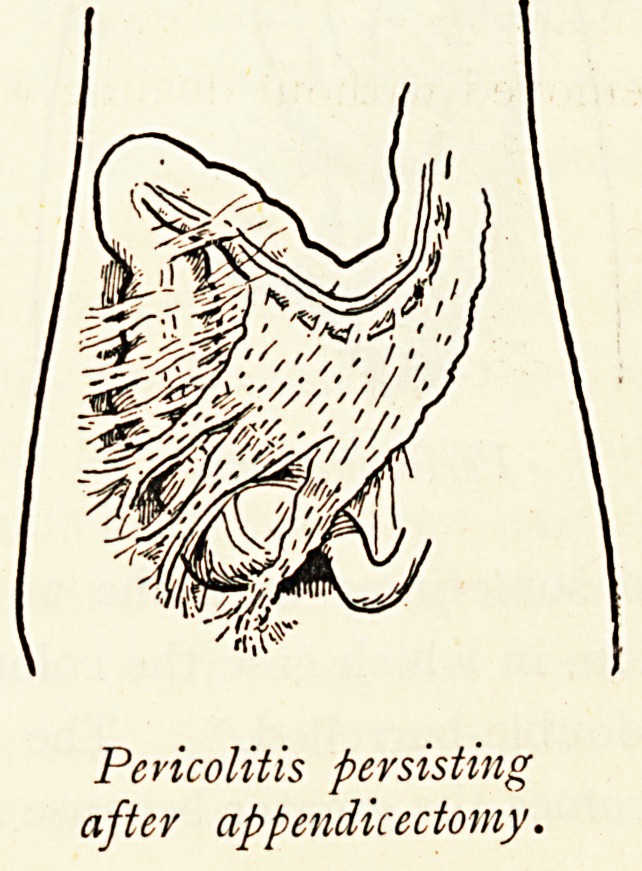


**Figure f6:**
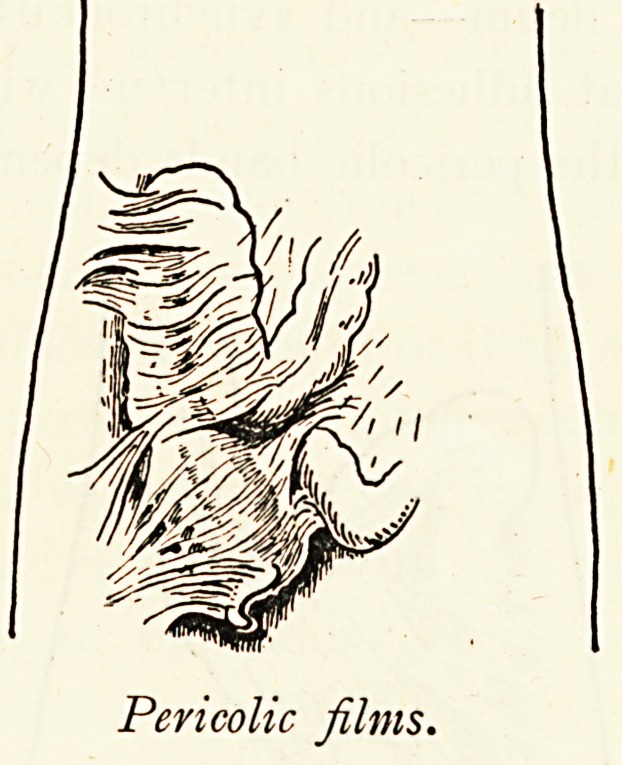


**Figure f7:**
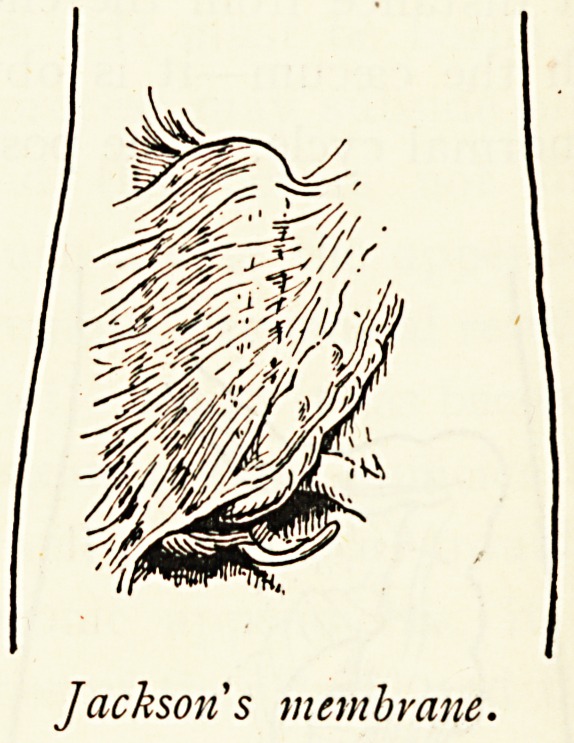


**Figure f8:**
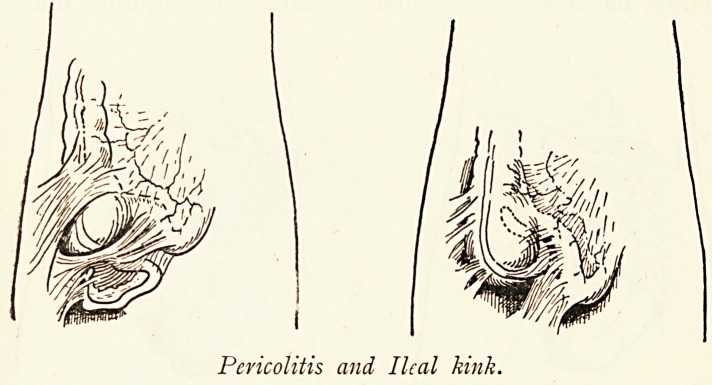


**Figure f9:**
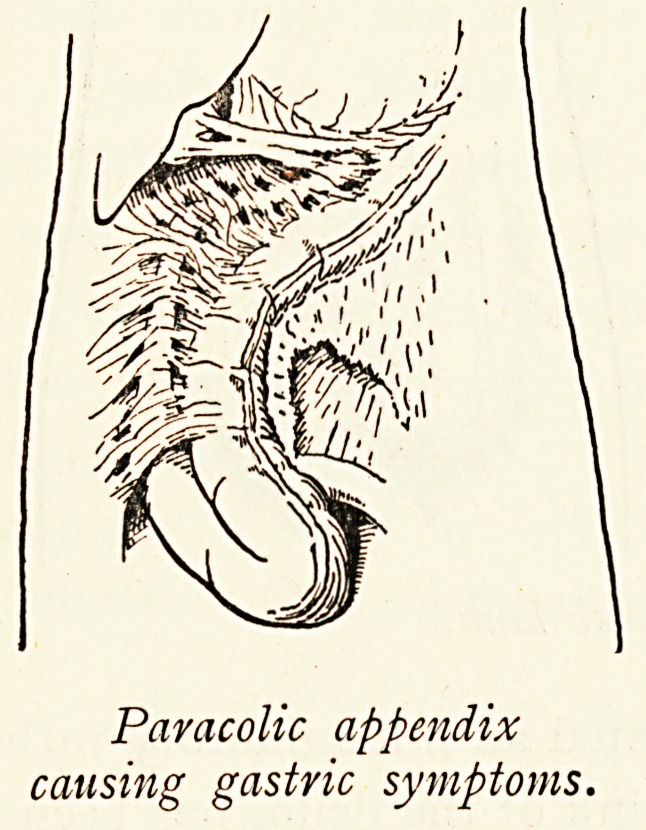


**Figure f10:**
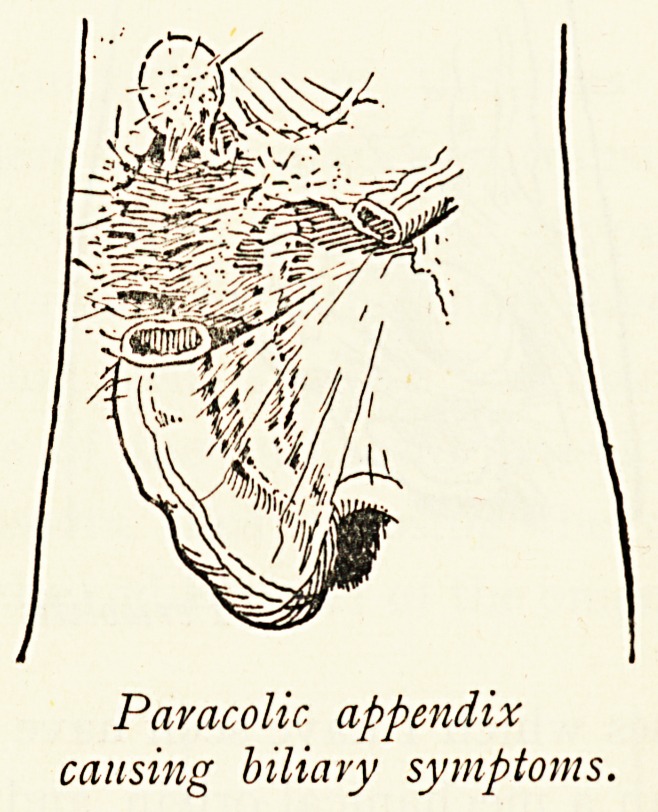


**Figure f11:**
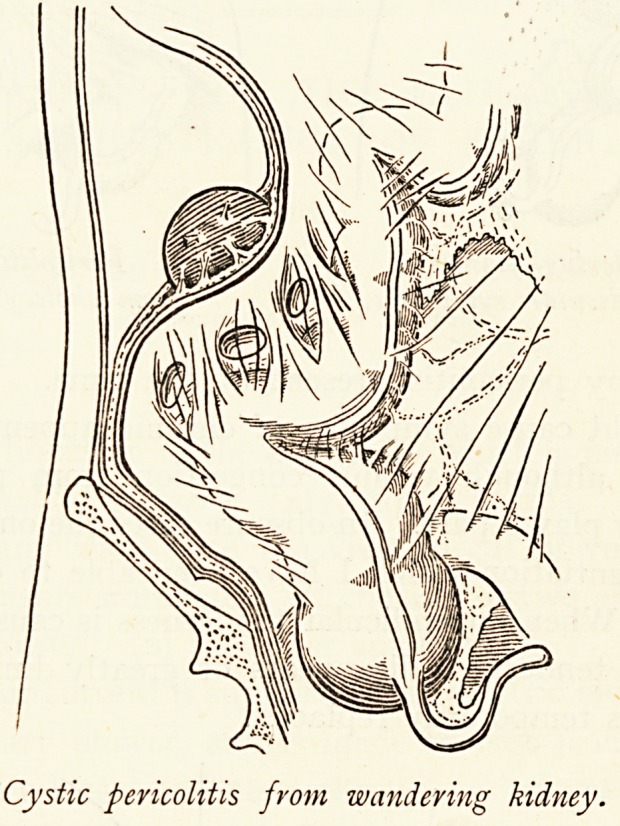


**Figure f12:**
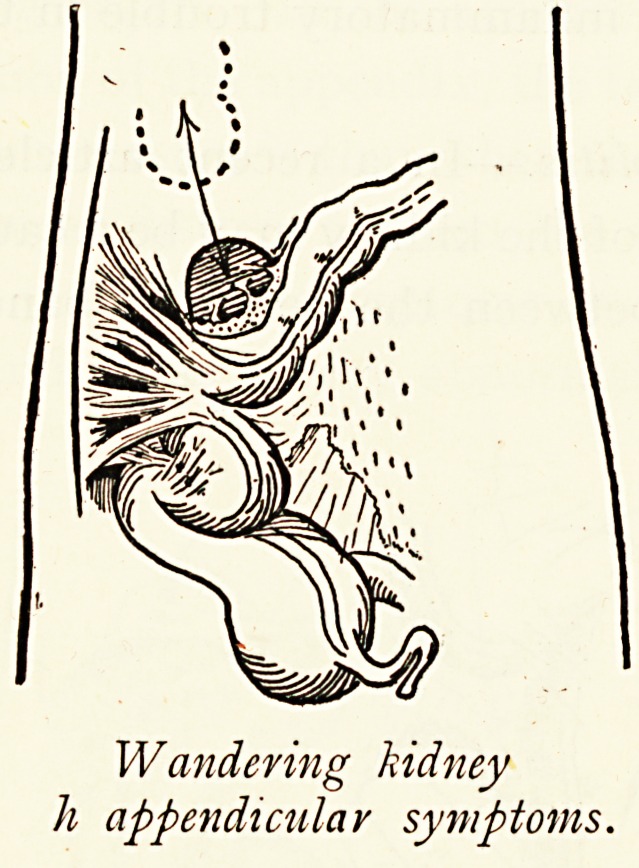


**Figure f13:**
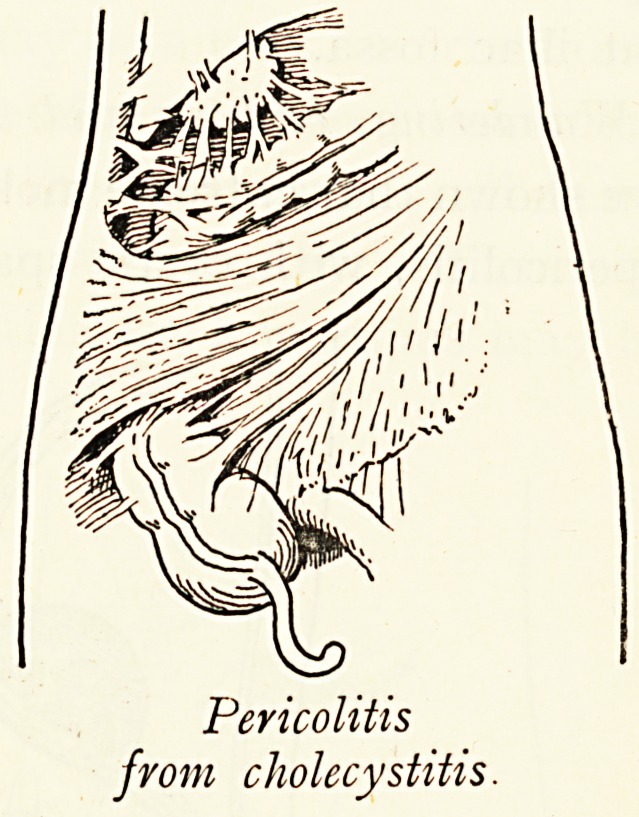


**Figure f14:**
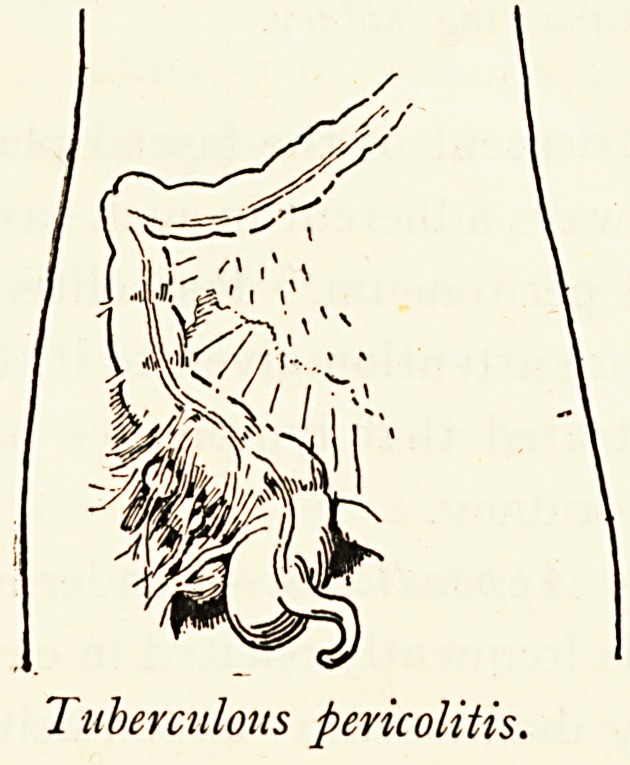


**Figure f15:**